# Active involvement of patients in pharmacist education has a positive impact on students’ perspective: a pilot study

**DOI:** 10.1186/s12909-020-02241-y

**Published:** 2020-09-21

**Authors:** Caroline Hache, Stéphane Honoré, Guillaume Hache

**Affiliations:** 1 Aix Marseille Univ, ADEF, 57 avenue escadrille Normandie Niemen, 13013 Marseille, France; 2 Aix Marseille Univ, Faculté de Pharmacie, Laboratoire de Pharmacie Clinique, 27, boulevard Jean Moulin, 13005 Marseille, France; 3 Aix Marseille Univ, APHM, Hôpital de la Timone, Service de Pharmacie, 264 rue saint Pierre, 13005 Marseille, France; 4 Aix Marseille Univ, INSERM, INRAE, C2VN, Faculté de Pharmacie, 27 boulevard Jean Moulin, 13005 Marseille, France

**Keywords:** Educational device, Patient educational training, Patient-led education, Pharmacy, University curriculum

## Abstract

**Background:**

Patient-led education contributes to the implementation of practical experience of working with patients in health care professional curricula. There are few descriptions of patients’ involvement in pharmacists’ training and most often, the patients have been used as passive props to facilitate training. More recently, greater emphasis has been given to a more active form of patient involvement but the application in the curriculum of pharmacy has not been conceptualized. Thus, the aim of our study was to implement a workshop involving patients as partners in undergraduate pharmacy educational programme, and to evaluate its impact of on students’ perspectives.

**Method:**

On a prospective observational study basis, the impact was assessed in terms of relevance, learning outcomes and achievement transfer using the Kirkpatrick training assessment method. In addition, we evaluated social representations of the students before and after the workshop.

**Results:**

Ninety-four students attended the sessions. All participants were satisfied and emphasized the relevance of the involvement of patients. Postworkshop scores were significantly improved in both competencies to be acquired. At the end of the workshop, students reported two to three actions to implement in order to meet patients’ expectations, illustrating an intent to transfer learning outcomes in professional context. Interestingly, about patients’ expectations on pharmacist’s role, students’ social representations had evolved significantly after the session.

**Conclusion:**

These results highlight the positive impact of the innovative workshops and the additive value of patients’ involvement in the pharmacy undergraduate programme.

## Background

Related to the emphasis of patient-centeredness in healthcare, there is an increasing promotion for public and patients’ involvement in education of health care professionals. Most of healthcare professionals in training are in favour of more direct patient involvement with their teaching [1], and it represents a challenge for healthcare professional educators in providing appropriate training. Patient-led education is the involvement of real patients, portraying their own experience of health care either personally or indirectly through their relatives [2]. Patient-led education can contribute to the implementation of practical experience of working with patients alongside the theoretical and scientific aspects of health care providers’ education.

During the last decade, pharmacy practices are evolving towards the concept of pharmaceutical care. Pharmaceutical care involves the process through which a pharmacist co-operates with a patient and other professionals in designing, implementing, and monitoring a therapeutic plan that will produce specific therapeutic outcomes for the patient [3]. Regarding pharmacists’ education, the general pharmaceutical council’s standards for pharmacy education in UK emphasizes this importance for future pharmacists [4]. Surprisingly, there are few descriptions of patients’ involvement in pharmacist training and most often, patients have been used as passive props to facilitate training, such as illustrations of interesting points. More recently, greater emphasis has been given to a more active form of patient involvement [5, 6]. Active involvement describes the involvement of people who are engaged in teaching and assessment. Active involvement of patients in health professional education is increasing, partly due to the recognition that patients have unique expertise derived from their experience of illness, disability or the effects of the social determinants of health [5].

Moreover, patients are able to transmit specific skills [7], and expert patients have been involved in the development of therapeutic patient education [8]. Therapeutic patient education enables people with chronic diseases to manage their illness and yields benefits in both health and financial terms. Therapeutic patient education is formalized in patient educational programmes (PEP). PEP must be provided by multidisciplinary teams of health care providers and should be part of the early training programme of every health care professional, including pharmacists. PEP emphasize a systemic, patient-centered learning process. While expert patients are key partners in the development of PEP, patients’ involvement in undergraduate programmes focusing on PEP is less documented in the literature. Unfortunately, theories which attempt to explain patients’ involvement are under-developed and have not been conceptualized in application to contextual settings [9].

Thus, the aims of our study was to implement a workshop involving patient partners during a module focusing on PEPs in undergraduate pharmacy programme, and to evaluate its impact on students’ perspectives.

## Methods

### Context

The study covered a module focused on PEP within the pharmacy undergraduate programme. In collaboration with four patients, we developed a workshop focused on two competencies to be acquired:

- Competency 1 (C1): to identify patients’ expectations about PEP
- Competency 2 (C2): to identify patients’ expectations towards pharmacist’ role in PEP.

This workshop lasted 2 hours and consisted of three different phases. First, we introduced the session with a round of introduction and the statements about the need for confidentiality and respect. Then, we announced competencies framework and explained why these competencies should be acquired for their professional development, in order to elaborate the learning agreement and illustrate the behaviours expected by the educational team [10].

Secondly, we performed an session inspired by the jigsaw classroom to create an interactive learning experience between patients and students, and to ensure the students’ involvement [11].

Following three steps:

- Step1- we defined random groups of students to explore one of the three following questions:
  - What are the patients’ expectations towards PEP?
  - What are the patients’ expectations towards pharmacists’ role specifically in PEP?
  - What are the patients’ expectations towards pharmacists’ role in general healthcare?

While, first questions were both exploring the two competencies to be acquired directly, the third question has been designed in order to discriminate specific expectations toward the role of pharmacists in PEP from their role in general healthcare.

One patient partner per group moderated, facilitated and illustrated the discussion, without any moderation from the faculty member for 20 min. Then, the patient left the group and students had 5 min to synthesize the discussion and identify learning outcomes from their discussion with the patient.

- Step2– we defined new groups of 4–5 students from different previous discussions, so that each student could share the previous outcomes with the others. This step is meant to identify all the learning outcomes generated from patients’ interviews, during the session. At the end of their discussion, they had to agree on one additive question or comment to address to the patients. During this step2, patients joined together to discuss about exchanges they had with students and agreed on two essential points to transmit for the conclusion.
- Step3- one student per group asked the questions/submit the comment to the entire class, in order to describe and clarify learning outcomes generated. This general feedback was moderated by the patients; the faculty was present in the room but did not intervene in the discussion.

Finally, patients concluded with their two essential items and the teacher summarized exchanges and comments discussed in collective.

### Assessment and measures

#### Impact on student perspectives

We estimated the impact of the workshops in terms of relevance, learning outcomes and achievement transfer [12, 13]. At the end of the sessions, students were invited to perform self-assessment questionnaires, in order to highlight:

- Relevance, by rating (1) the level of satisfaction; (2) the level of learning “I learn”; (3) the level of transferability “I think I may be able to use into (professional) context”;
- Learning outcomes from C1 and C2, by (i) self-rated scales (from 1 to 5) about perceived knowledge of these topics, both before and after the workshops. *(*“*For each item, please estimate your level of knowledge (before/after the session): (1) Patients’ expectations about PEP; (2) Patients’ expectations about pharmacist’ role in PEP.*”*)*, and (ii) an open question about what they learnt;
- Achievement transfer, by asking students to write skills they may be able to use into professional context.

#### Social representations

The conception of social representations have been suggested as a contributor to the explanation of learning processes [14, 15]. The origins of the social representations theory are attributed to Moscovici [16]. Social representations are described as a mental phenomenon, with both a structure and contents, which integrates perception and understanding concepts [17]. From the spontaneous expression of individuals’ knowledge, it aims to estimate common knowledge and to highlight mental products created by a community of human life. In this theory, social representations result from social interactions and discussions [18], and evolve in social interactions everyday. Thus, analysing social representations provide an opportunity to identify individuals’ knowledge about a complex object, and the way it is organized and used by a group [19]. It contributes to interpret and understand a given social context [20]. Moreover, social representations may have a strong impact on learning process because, depending on the nature of the learning object, knowledge to be acquired in a classroom may be challenged by the knowledge previously acquired by the students outside the formal instructional context. In a learning context, it is important to consider knowledge acquisition as a product of formal, informal and nonformal learning. Formal learning is the kind of learning designed in formal contexts, such as the workshop. Informal and nonformal learning takes place everywhere, through all kinds of means. The connection between these different forms of learning emphasizes the role people’s social representations play in the results of learning [14].

Social representations are brought out by collecting free associations produced from an inductive sentence [21, 22]. The whole social representation is organised by a limited number of largely shared and consensual elements. This limited number of cognitions is called the central core of social representations. Around the central core, there are many peripheral elements depending on the weighting, the value or the function assigned to it. These elements perform several functions, such as regulating and protecting the central core, thus making it concrete [23]. In this workshop, the sentence was “pharmacists’ role in PEP”. We gave the instruction: “write three words, expressions or adjectives which spontaneously come to your mind when you hear the sentence ‘pharmacists’ role in PEP’” [24]. Then, we asked the participants to rank their words from the most important (= 1) to the least important word (= 3) [23]. In order to estimate the influence of the workshop involving patient partners on the perception of students about patients’ expectations toward PEP, we chose to identify the social representations before the session, and to compare them to what was expressed at the end the workshop.

### Analysis of the data

Quantitative data were analyzed statistically using a Wilcoxon matched-pairs signed rank test for repeated measures. For the analysis of social representations, we used the rank /frequency method in order to identify the structure of the social representations of the group about pharmacists’ role in PEP, as previously described [23, 24]. Briefly, words were distributed from either sides of mean rank and mean frequency into four sets (Table 1) as follow: the central core integrates high frequency associations which are ranked as important by the subjects; the first periphery integrates high frequency associations which are ranked as less important by students, and illustrates the central core; the contrasted zone integrates associations with low frequency and high rank, and may be considered as elements which complete the first periphery, or either reveal the existence of a subgroup that shares a different but strong social representation; and the second periphery integrates associations, with a low rank and a low frequency, which illustrate elements of second importance for the students [24].

**Table 1:** Identification of the structure of social representations

	High rank (≤ mean of average rank)	Low rank (> mean of average rank)
High frequency (≥ mean frequency)	**Central core**	**First periphery**
Low frequency (< mean frequency)	**Contrasted elements**	**Second periphery**

### Ethics

The pedagogical committee of Aix-Marseille University-School of Pharmacy that deals with research authorizations and ethical considerations in the field of education has approved the study. Verbal consent was obtained from study participants and approved by the committee. The ethical considerations taken into account were based on the principles outlined in the Declaration of Helsinki.

## Results

Ninety-four students attended the session, 24 men and 70 women, and they were between 20 and 23 years old. Sex ratio and age are representative of pharmacist students in France [25].

### Impact on student perspectives

**Fig. 1:**
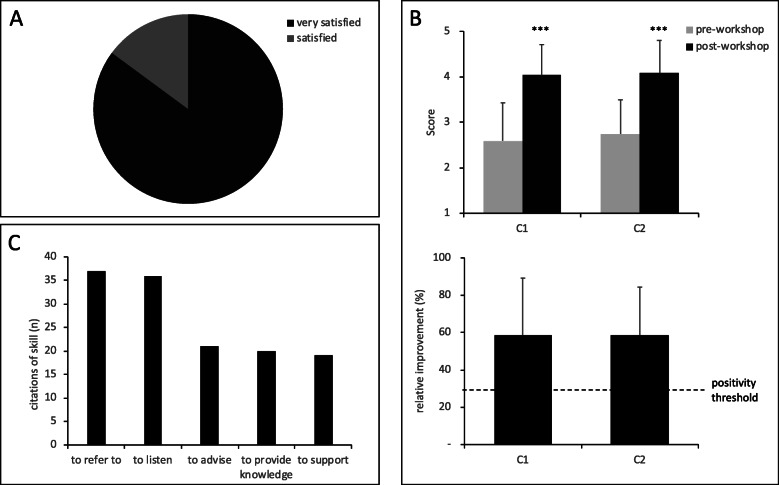
The impact of the workshop assessed by the kirkpatrick training assessment method. Relevance (**a**), learning outcomes (**b**) and achievement transfert (**c**) are the three learner-related impact markers. Learning outcomes data are plotted as mean ± SD. *** *p* < 0.001 versus pre-workshop

All participants considered themselves “satisfied” or “very satisfied” (Fig. 1a), and emphasized the relevance of the involvement of the patients in this session. “The involvement of patients” and “interaction/sharing/discussion” were expressed in the majority of the evaluation charts, with 93 and 72% of citations, respectively. Specifically, they expressed their satisfaction to the questions “What did you like during the workshop? ” in the self-assessment questionnaire:

*To identify patients’ perspectives who are directly affected, to be part of the workshop, to exchange*

*No theoretical course but active involvement/team work with real patients. All of our questions are about to be answered.*

*Patients’ involvement, the opportunity to interact in small groups, to compare our theory with patient expectation*

*The involvement of patients partners makes the course more specific/concrete*

One of the verbatim was really important because it highlighted the difficulty for students, in their position of trainee during an internship at the hospital or in a community pharmacy:

*Direct contact with patients, without any professional barrier.*

Post-workshop scores were significantly improved for C1 (4.0 ± 0.7 vs. 2.6 ± 0.8; *p* < 0.001) and C2 (4.1 ± 0.7 vs. 2.7 ± 0.7; *p* < 0.001), highlighting increases in perceived knowledge about skills. Relative improvement of 58 ± 31% and 59 ± 25% per student, respectively, illustrate the effectiveness of teaching (Fig. 1b).

Besides the self-assessment, the students reported having learnt “the reflection on the place and the role of the pharmacist, specifically about skills of listening, empathy and humanity” (66/94; 70%), “the importance of PEP for patients” (31/94; 33%), “the importance to refer patients to” (22/94; 23%), “the role of the pharmacist in a medical team” (39/94; 42%), “identifying patients’ expectations” (43/94; 46%). In more details, some students expressed specific learning outcomes:

*I have further integrated [...] that listening is the most important, with empathy. Advices expected by the patients are not necessarily related to medications. The importance of patients associations ++*

*I’ve learned that patients are very interested in the social aspect of health care. I could feel their experience.*

*I learned to put myself in the shoes of a patient, and to be able to observe my pharmacist’s role through patient’s eyes.*

*We need to focus on the expectations of each patient, not just use a general approach but to tailor it to a patient.*

*My human qualities were even more essential than my professionnal knowledge as a pharmacist*

*I’ve learned that patient’s view of us may not be what I thought it was.*

*I am more aware of what the patient is expecting from the community pharmacy.*

Students reported between two and three actions to be implemented in order to meet patients’ expectations. The most cited actions were “to refer” (−to patients’ associations, −to PEP, −to a psychologist) (37/94; 39%), “to listen” (36/94; 38%), “to advise”(21/94; 22%), “to provide (expert) knowledge” (5/32; 15.6%) and “to support” (19/94; 20%) (Fig. 1c). Students expressed important actions they plan to implement in their professional activities:

*Information and advice beyond medication supply. Listening. To be involved in prevention. Use language that everyone can understand.*

*Thanks to this workshop, I will be more able to exploring the expectations of the patients, and subsequently advice and support them efficiently.*

*To implement individual PEP sessions in pharmacies. In any case, take the time to know the patient[...]. Listen, adapt and follow up.*

*To listen and advise my patients while respecting their individuality. I would like to make PEP a large part of my job so this type of session is a rewarding experience for me.*

### Structure of students’ social representations about pharmacist’s role in PEP

Before the workshop, the central core was composed of two elements: to listen and compliance. The first periphery was composed of four elements: to support, to provide (expert) knowledge, to explain and to advise. Contrasted elements were composed of to understand, trust/confidence and to reassure. Interestingly, we identified some changes in social representations between before (Table 2) and after (Table 3) the workshop. As a matter of fact, trust/confidence raised from contrasted element to the central core, and to reassure raised from contrasted elements to the first periphery; empathy was a new association in the central core and compliance disappeared.

**Table 2:** Structure of social representations before the workshop

BEFORE WORKSHOP		
	High rank (≤ 1.96)	Low rank (> 1.96)
High frequency (≥ 12.71)	**Central core**	**First periphery**
	To listen (38; 1.4) Compliance (17; 1.8)	To support (54; 2) To provide (expert) knowledge (40; 2.2) To explain (18; 2.2) To advise (16; 2.1)
Low frequency (< 12.71)	**Contrasted elements**	**Second periphery**
	To understand (9; 1.4) Trust/confidence (5; 1.8) To reassure (4; 1.8)	To help (12; 2.2) Communication (11; 2.0) To educate (10; 2.2) Follow-up (6; 2.7) To share (6; 2.2)

**Table 3:** Structure of social representations after the workshop

AFTER WORKSHOP		
	High rank (≤ 1.98)	Low rank (> 1.98)
High frequency (≥ 11.27)	**Central core**	**First periphery**
	To listen (45; 1.5) Empathy (20; 1.8) Trust/confidence (17; 1.6)	To provide (expert) knowledge (45; 2.2) To support (44; 2.1) To advise (32; 2.2) To reassure (15; 2.1)
Low frequency (< 11.27)	**Contrasted elements**	**Second periphery**
	Humanity (8; 1.4) To help (6; 1.5) Integrated consideration (4; 1.8)	To dialogue/interact (9; 2.3) To explain (9; 2.2) To refer (7; 2.6) To educate (5; 2.0)

We compared the changes of social representation before and after the workshop depending on the gender of students. Before the workshop (Table suppl. 1), the central core expressed by men consisted of three elements whereas women had only one strong element: to listen. When we consider the central core and the first periphery of both, only two elements are different: to advise for women and compliance for men. After the workshop (Table suppl. 2), the element empathy appeared in the central core of men and women. We found the elements trust/confidence and to reassure only in women central core and first periphery.

## Discussion

In this report, we describe an innovative workshop for students in Pharmacy, patient-centered and focused on professional skills. We chose to integrate this workshop within a module about PEP, because PEP emphasized patient roles and expertise by involving patient partners alongside of the process [26]. The first innovation was to adapt the jigsaw classroom as an alternative to traditional classroom teaching methods, by organizing students in smaller groups so that they learnt by relying on each other rather than the teacher [11]. In addition, we involved patient partners during the session in order to integrate patients’ perspectives in the learning process. Thus, their experience in terms of gender, age, illness trajectory and role as patient emphasized the individual nature of the illness experience and the need for care to be personalized [5]. Patient engagement is a promising approach to improve population’s health and the quality of services provided by the health system. During the last decade, the University of Montreal has conceptualized a model based on the partnership between patients and healthcare professionals, so called the Montreal model. This model suggest a continuum of patient engagement in various domains, namely direct care, organisation of services/governance, health policies, research and teaching [27]. According to this model, the active participation of patient during our workshop covered most of the “collaborative” level of engagement in teaching, because of their training and specific tasks assigned during the workshop. Moreover, they joined teaching with experience-based knowledge-sharing during the session. Thus, our workshop covered some elements of the “partnership” level suggested by the theoretical framework of the Montreal model. To our knowledge, we provide the first report providing practical information about implementing the jigsaw classroom, in partnership with real patients, in the curriculum of pharmacy. This was possible through a close collaboration between patients, researchers and pharmacists.

Our results demonstrate the positive impact of this innovative workshop on students’ perspectives in undergraduate pharmacy training programme (Fig. 1). Workshops were well received by students, a finding consistent with most other reports about patient involvement [5]. Students’ satisfaction and will to develop new skills illustrate their interest towards their involvement in PEP. Significant increases in post-workshop scores demonstrate that participants felt they acquired knowledge, which is a metacognitive approach of learning [28]. Metacognition, which is the awareness of procedures, methods and intellectual processes implemented to learn, contributes to acquisition of knowledge [29]. In addition, the relative improvement (> 50%) for each skill highlights the teaching effectiveness of the session and contributes to the validation of our educational framework [13].

Assessing competencies acquired by the students was challenging as behavioural evolution is difficult to explore in such framework. However, all participants reported intention to set up actions in their professional context in order to meet patients’ expectations. Although virtual, these results suggest that students were not reluctant to changes, and that they felt able to improve their behaviours. Perceived self-efficacy is defined as people’s judgements of their capacities to organize and execute course of action required to attain designated types of performances [30] and our results highlight a high level of self-efficacy after the workshop. Bandura proposed that perceived self-efficacy was related to performance, and a meta-analysis supports that perceived self-efficacy is related to both performance and behaviour choice [31]. More recently, self-efficacy has been identified as a motivational parameter involved in both adoption of-, and long-term adherence to these behaviours [32, 33]. Thus, we suggest that our results about self-efficacy are promising regarding the students’ ability to implement the competencies in their professional context.

The involvement of patient partners was the cornerstone of the development and the implementation of this workshop. Patients’ involvement has been the most appreciated aspect of the workshop by the students. As a matter of fact, “the involvement of patients” and “interaction/sharing/discussion” were expressed in the majority of evaluation charts. The students pointed out that the structure of the workshop had made it possible to discuss, exchange with patients freely on their feelings and experiences. It emphasizes the relevance of patients’ involvement in the device. Interestingly, the most cited skill to be set up in a professional context was “to refer to another institution or professional or association”. This skill was not mentioned in social representations before the session but appeared after the session, in the second periphery (Table 3). We must underlie that this skill was spontaneously expressed by the patients about their expectations for pharmacists’ role, without any suggestion from the faculty member. This example illustrates the impact of patients’ involvement on students’ learning outcomes. It may also illustrate that the take home message is very dependent of the diversity of patient partners. These concepts form the foundation of patient-centered care, and are concepts that students rarely learn in classes, where similarities rather than diversity among patients with a particular disease are emphasized [5, 34].

The conception of social representations have been suggested as a contributor to the explanation of the learning processes [14, 15], so we explored social representations of the students on pharmacists’ role in PEP. In this learning context, the central idea was that people already had some representations of the learning object prior to the engagement in any formal learning process, and all teaching and all learning proceeds from pre-existent knowledge [35]. Thus, we chose to assess social representation at the beginning of the workshop in order to identify students’ pre-existent knowledge, and to further work with the students integrating these representations (Table 2). We also sought for social representations after the workshops and highlighted that students’ social representations changed. Importantly, the central core constitutes the fundamental element which organizes the representations and characterizes the social object [36], and “compliance” disappeared from the central core in our study. Compliance is defined as the extent to which the patient’s actual history of drug medication corresponds to the prescribed regimen [37], which convey the idea of obedience to a prescription [38] and passive behaviour from patients. Its vanishing from social representations suggests that students realized the patients did not expect to be passive in their relationship with the pharmacist. This illustrates that patients can convey crucial notions of pharmaceutical care to pharmacy students. In this regard, “empathy” and “trust/confidence” emerged in the central core after the session, highlighting that students identified the complementarity between soft skills and professional expertise. Moreover, the first periphery slightly evolved as well, and “to reassure” replaced “to explain”. This part of the social representations has a defensive function of the core, and an adaptive function to allow adjustments [39]. These changes illustrated a reorganization of the representations [23]. Finally, news words appeared on contrasted element such as “humanity” and “consideration”. Taken together, these results suggest that students focused more on self-attitudes and -competencies rather than “compliance”. These profound modifications may have resulted from messages communicated by the patients during the workshop, and illustrate that credible sources had a conclusive impact on the structure of the social representations [26].

More specifically, we compared the changes in social representations between men and woman. We note that there is no gender differences concerning the apparition of “empathy” in the central core. After the workshop, men and women considered this soft skill as important. It is suggested in the literature that women are more empathic than men [40, 41]. According to our result, men and women tend to be equally sensitive to empathy in the relationship with the patient after the workshop. However, women express more soft skills than men in their social representation, such as “to reassure” in the first periphery and “integrated consideration” in the contrasted elemens. These results could indicate that, compared to men, women are more sensitive to soft skills in general.

The social representations are a determinant of behaviours and practices [36, 42, 43]. The changes in representations between the beginning and the end of the workshop, associated with the impact highlighted by crossing relevance, learning outcomes and achievement transfer, suggests that the behaviour of students may further change. Unfortunately, our study was not designed to assess effective skill in professional context, and this should be assessed in further studies. Notwithstanding, our results illustrated that the students focused towards the acquisition of professional skills and attitudes, expected by patients. This is consistent with the new competency-based paradigm [44] promoted for university teaching.

## Conclusion

This study highlights the feasibility and the effectiveness of the implementation of a patient-led workshop in the pharmacy undergraduate programme. Our result suggests positive impact on students’ perspectives in terms of satisfaction, acquisition of knowledge and intent to transfer.

## Supplementary information

**Additional file 1: Table suppl.1:** Gender related structure of social representations before the workshop. **Table suppl.2:** Gender related structure of social representations after the workshop.

## Data Availability

The datasets used and/or analysed during the current study are available from the corresponding author on reasonable request.

## Abbreviations

PEP: Patient educational programmes
C1: Competencie 1
C2: Competency 2
