# Supplementary material for: Active involvement of patients in pharmacist education has a positive impact on students’ perspective: a pilot study
Source: BMC Med Educ. 2020 Sep 21;20:322. doi: 10.1186/s12909-020-02241-y (PMC7507651; doi:10.1186/s12909-020-02241-y)
Supplement: Supplementary file 1 — Additional file 1: Table suppl.1: Gender related structure of social representations before the workshop. Table suppl.2: Gender related structure of social representations after the workshop. [file 12909_2020_2241_MOESM1_ESM.docx]

**SUPPLEMENTAL MATERIAL**

Table suppl.1 : Gender related structure of social representations before the workshop

| \| **For men** \| \| \| \| --- \| --- \| --- \| \|  \| High rank (≤ 1.95) \| Low rank (> 1.95) \| \| High frequency  (≥ 4.43) \| **Central core** \| **First periphery** \| \| To support (10; 1.8)  To listen (8; 1.4)  Compliance (6; 1.7) \| To provide (expert) knowledge (12; 2,3)  To advise (5; 2.2) \| \| Low frequency  (< 4.43) \| **Contrasted elements** \| **Second periphery** \| \|  \| Communication (3; 2.3)  To explain (3; 2.3)  To follow up (2; 2,3)  To help (2; 2)  Trust/Confidence (2; 2)  To reassure (2; 2)  To share (2; 2) \| | \| **For women** \| \| \| \| --- \| --- \| --- \| \|  \| High rank (≤ 1.98) \| Low rank (> 1.98) \| \| High frequency  (≥ 10.94) \| **Central core** \| **First periphery** \| \| To listen (30; 1.5)  Compliance (11; 1.8) \| To support (44; 2)  To provide (expert) knowledge (28; 2.2)  To explain (15; 2.1)  To advise (11; 2) \| \| Low frequency  (< 10.94) \| **Contrasted elements** \| **Second periphery** \| \| To understand (9; 1.4)  Communication (8; 1.9)  Trust/confidence (3;1.7)  To reassure (2;1.5) \| To help (10; 2.3)  To educate (10; 2.2)  To follow-up (4; 3)  To share (4; 2.2) \| |
| --- | --- | --- | --- | --- | --- | --- | --- | --- | --- | --- | --- | --- | --- | --- | --- | --- | --- | --- | --- | --- | --- | --- | --- | --- | --- | --- | --- | --- | --- | --- | --- | --- | --- |

Table suppl.2 : Gender related structure of social representations after the workshop

| \| **For men** \| \| \| \| --- \| --- \| --- \| \|  \| High rank (≤ 1.99) \| Low rank (> 1.99) \| \| High frequency  (≥ 6.18) \| **Central core** \| **First periphery** \| \| To listen (13; 1.5)  Empathy (7; 1.8) \| To support (13; 2.3)  To advise (10; 2.3)  To provide (expert) knowledge (10; 2.1) \| \| Low frequency  (< 6.18) \| **Contrasted elements** \| **Second periphery** \| \| Trust/confidence (5; 1.6) \| To explain (4; 2.5)  To reassure (3; 2)  To dialogue/interact (3; 2)  To refer (1; 2) \| | \| **For women** \| \| \| \| --- \| --- \| --- \| \|  \| High rank (≤ 1.98) \| Low rank (> 1.98) \| \| High frequency  (≥ 10.19) \| **Central core** \| **First periphery** \| \| To listen (32; 1.5)  Empathy (13; 1.8)  Trust/confidence (12; 1.6) \| To provide (expert) knowledge (35; 2.4)  To support (31; 2)  To advise (22; 2.1)  To reassure (12; 2.2) \| \| Low frequency  (< 10.19) \| **Contrasted elements** \| **Second periphery** \| \| Humanity (8; 1.4)  To help (6; 1.5)  Integrated consideration (4; 1.8) \| To dialogue /interact (6; 2.5)  To refer (6; 2.7)  To explain (5; 2)  To educate (5; 2) \| |
| --- | --- | --- | --- | --- | --- | --- | --- | --- | --- | --- | --- | --- | --- | --- | --- | --- | --- | --- | --- | --- | --- | --- | --- | --- | --- | --- | --- | --- | --- | --- | --- | --- | --- |
